# Dexmedetomidine administration reduced mortality in patients with acute respiratory distress syndrome: a propensity score-matched cohort analysis

**DOI:** 10.3389/fmed.2025.1565098

**Published:** 2025-04-17

**Authors:** Conglin Ye, Yang Yu, Yi Liu

**Affiliations:** ^1^Department of Critical Care Medicine, The First Affiliated Hospital of Gannan Medical University, Ganzhou, Jiangxi, China; ^2^The First Clinical Medical College of Gannan Medical University, Ganzhou, Jiangxi, China

**Keywords:** dexmedetomidine, acute respiratory distress syndrome, mortality, propensity score, MIMIC IV database

## Abstract

**Background:**

Acute respiratory distress syndrome (ARDS) continues to pose significant difficulties due to the scarcity of successful preventative and therapeutic measures. Recent clinical trials and experimental research have confirmed the lung-protective and anti-inflammatory properties of dexmedetomidine. The objective of this study was to examine the relationship between the use of dexmedetomidine and mortality outcomes in ICU patients with ARDS.

**Methods:**

This study retrospectively examined data from the Medical Information Mart for Intensive Care (MIMIC) IV, focusing on individuals diagnosed with ARDS. The primary endpoint was the occurrence of death within 28 days after entering the ICU. To ensure a balanced cohort, we applied propensity score matching at a 1:1 ratio. Additionally, multivariable analysis was performed to mitigate the effects of confounding factors.

**Results:**

In this study, a cohort comprising 612 patients diagnosed with ARDS was investigated. Analysis using both univariate and multivariate Cox regression indicated significantly reduced 28-day and 90-day mortality rates in patients administered dexmedetomidine compared to those who were not given this treatment. Following adjustments for potential confounders using propensity score matching, these results were confirmed to be robust.

**Conclusion:**

The results indicate an association between the administration of dexmedetomidine and lower mortality rates among severely ill ARDS patients. However, this result should be interpreted with cause because of a lot of missing data of potential risk factors for clinical outcomes. Nonetheless, it is imperative to perform further randomized controlled trials to corroborate this finding.

## 1 Introduction

ARDS is identified as an advanced stage of respiratory failure, prominently featuring marked hypoxemia alongside clearly visible bilateral infiltrates on radiographic scans (1–4). Annually, around three million individuals worldwide receive a diagnosis of ARDS, representing nearly 10% of admissions to Intensive Care Units (ICUs) (5–7). Notwithstanding significant therapeutic progress, the mortality rates linked to ARDS have largely persisted since the late 1990s (8, 9). Current research estimates in-hospital mortality rates for ARDS to be between 34.9 and 46.1% (5). The pathophysiology of ARDS is intricate, encompassing the activation and dysregulation of various injury response pathways, alongside inflammatory and coagulation processes that influence both pulmonary and systemic circulation (10). These complexities highlight the pressing necessity for innovative and efficacious therapeutic approaches to enhance clinical outcomes and alleviate the health burden linked to ARDS.

Dexmedetomidine (DEX), extensively used as a sedative in intensive care units, has drawn rising attention because of its anti-inflammatory, immunomodulatory, antioxidant and organ protective properties. According to research in animal models, DEX reduces lung injury because of its multiple protective properties (11, 12). In addition, DEX has also been demonstrated to have protective effects against renal and cardiac ischemia and reperfusion injuries (13, 14). Clinical trials show that DEX decreases inflammation and assists in retaining renal and pulmonary functions during surgery (15). Despite the growing evidence of DEX’s efficacy in reducing inflammation and protecting organs, there remains a noticeable lack of specific clinical trials assessing its effectiveness in improving outcomes for patients with severe ARDS. To fill this gap, a thorough retrospective cohort study was started to examine the effect of DEX treatment on the mortality rates of patients who suffered this type of respiratory failure.

## 2 Method

### 2.1 Data source

All research data we used were from the MIMIC-IV database, release version 3.1 (16). MIMIC-IV is a complete collection of patient information from electronic health records within Beth Israel Deaconess Medical Center at Boston, Massachusetts, USA. It consists of rich demographic profiles, vast laboratory test results, complex treatment regimens, and subtle intensive care monitoring records (17). Only researchers who have successfully participated in specialized training courses concerning ethical considerations and protection of human subjects in research have access to this repository. Prior to engaging in data analysis, the investigator CLY was granted the requisite permissions to utilize these resources (certification number: 36,937,255).

### 2.2 Study population and data extraction

The dataset for this study was sourced from the MIMIC-IV database, which provided comprehensive hospital admission records. The identification of patients suffering from ARDS was accomplished through specific ICD-9 codes: J80 and R0603. The criteria for exclusion included any patient whose ICU duration was less than 72 h or who was younger than 18 years of age at the time of admission. In instances where a patient had multiple ICU admissions, the analysis was restricted to only the first ICU stay. Within the cohort, patients who were administered dexmedetomidine (DEX) during their ICU admission were grouped as the DEX cohort, and those who did not receive DEX were designated as the non-DEX cohort.

### 2.3 Outcomes

The principal outcome of this study was mortality within 28 days following ICU admission. The secondary outcome evaluated was the mortality rates at 90 days after the admission to the ICU.

### 2.4 Data analysis

Continuous variables are described using the median alongside the interquartile range (IQR), while categorical variables are summarized as counts and percentages. The Kolmogorov–Smirnov test was applied to examine the distribution of continuous data for normality. For non-normally distributed continuous variables, the Wilcoxon rank-sum test was used. The Pearson’s chi-square test was performed for comparing categorical data.

The association between DEX administration and mortality risk was analyzed using Cox proportional hazards regression models, generating hazard ratios (HRs) with 95% confidence intervals (CIs). Kaplan–Meier survival analysis was utilized to evaluate survival distributions, and comparisons were made using the log-rank test. A multivariable Cox proportional hazards regression model was further applied to assess the relationship between DEX use and mortality outcomes. To address potential confounders, baseline characteristics of patients treated with DEX and those who were not were balanced using Propensity Score Matching (PSM). A 1:1 nearest-neighbor matching approach with a caliper of 0.02, performed without replacement, was utilized for this purpose. The variables included in Table 1 were used to estimate propensity scores. Both the primary and secondary outcomes were re-evaluated within the matched cohort. Statistical analyses were carried out using SPSS software (version 27.0, IBM, USA), and statistical significance was defined as *p* < 0.05.

**Table 1:** Baseline characteristics of ARDS patient cohorts pre- and post-matching.

Clinical variable	Before PSM				After PSM			
	Total	Non-DEX	DEX	*p*-value	Total	Non-DEX	DEX	*p*-value
	*n* = 612	*n* = 232	*n* = 380		*n* = 348	*n* = 174	*n* = 174	
Age	61.7 (50.1, 71.0)	65.0 (55.2, 72.7)	59.5 (47.7, 70.0)	**<0.001**	63.9 (52.1, 71.2)	64.2 (53.1, 70.1)	63.6 (51.3, 73.0)	0.585
Sex (female)	249 (40.7%)	104 (44.8%)	145 (38.2%)	0.103	154 (44.3%)	80 (46.0%)	74 (42.5%)	0.517
Race (white)	269 (44.0%)	97 (41.8%)	172 (45.3%)	0.404	139 (39.9%)	64 (36.8%)	75 (43.1%)	0.229
CHF	120 (19.6%)	52 (22.4%)	68 (17.9%)	0.172	70 (20.1%)	35 (20.1%)	35 (20.1%)	1.0
Sepsis	565 (92.3%)	197 (84.9%)	368 (96.8%)	**<0.001**	330 (94.8%)	166 (95.4%)	164 (94.3%)	0.628
Hypertension	217 (35.5%)	79 (34.1%)	138 (36.3%)	0.57	118 (33.9%)	60 (34.5%)	58 (33.3%)	0.821
Diabetes	202 (33.0%)	70 (30.2%)	132 (34.7%)	0.244	105 (30.2%)	53 (30.5%)	52 (29.9%)	0.907
COPD	138 (22.5%)	54 (23.3%)	84 (22.1%)	0.737	74 (21.3%)	38 (21.8%)	36 (20.7%)	0.793
AKI	598 (97.7)	226 (97.4)	372 (97.9)	0.699	342 (98.3)	170 (97.7)	172 (98.9)	0.410
SOFA score	7 (4.0, 10.0)	7 (3.0, 10.8)	7 (4.0, 9.8)	0.862	8 (4.0, 10.0)	8 (3.0, 11.0)	7.5 (4.8, 10.0)	0.944
APS III	55.0 (42.0, 74.0)	56.0 (43.0, 76.0)	54.0 (42.0, 69.0)	0.390	56.5 (46.0, 77.8)	59.0 (46.0, 77.3)	54.5 (46.0, 78.5)	0.675
Heart rate	93 (80, 108)	92 (78, 106)	93 (80, 108)	0.398	92 (79, 108)	93 (78, 109)	92 (80, 108)	0.869
MBP	84.0 (74.0, 95.0)	84.5 (73.3, 95.0)	84.0 (74.0, 95.0)	0.851	83.0 (73.0, 93.0)	84.0 (73.0, 92.0)	83 (73.0, 94.0)	0.683
Respiration rate	24 (20, 28)	24 (20, 29)	24 (19, 28)	0.505	24 (20, 29)	24 (20, 29)	24 (20, 29)	0.856
CRRT	187 (30.6%)	78 (33.6%)	109 (28.7%)	0.198	131 (37.6%)	65 (37.4%)	66 (37.9%)	0.912
Invasive ventilator	532 (86.9%)	165 (71.1%)	367 (96.6%)	**<0.001**	321 (92.2%)	160 (92.0%)	161 (92.5%)	0.841

CHF, congestive heart failure; COPD, chronic pulmonary disease; AKI, acute kidney injury; SOFA score, sequential organ failure score; APS III, acute physiology score III; MBP, mean arterial blood pressure; CRRT, continuous renal replacement therapy. Bold values are less than 0.05.

## 3 Results

### 3.1 Patient characteristics

**Figure 1:**
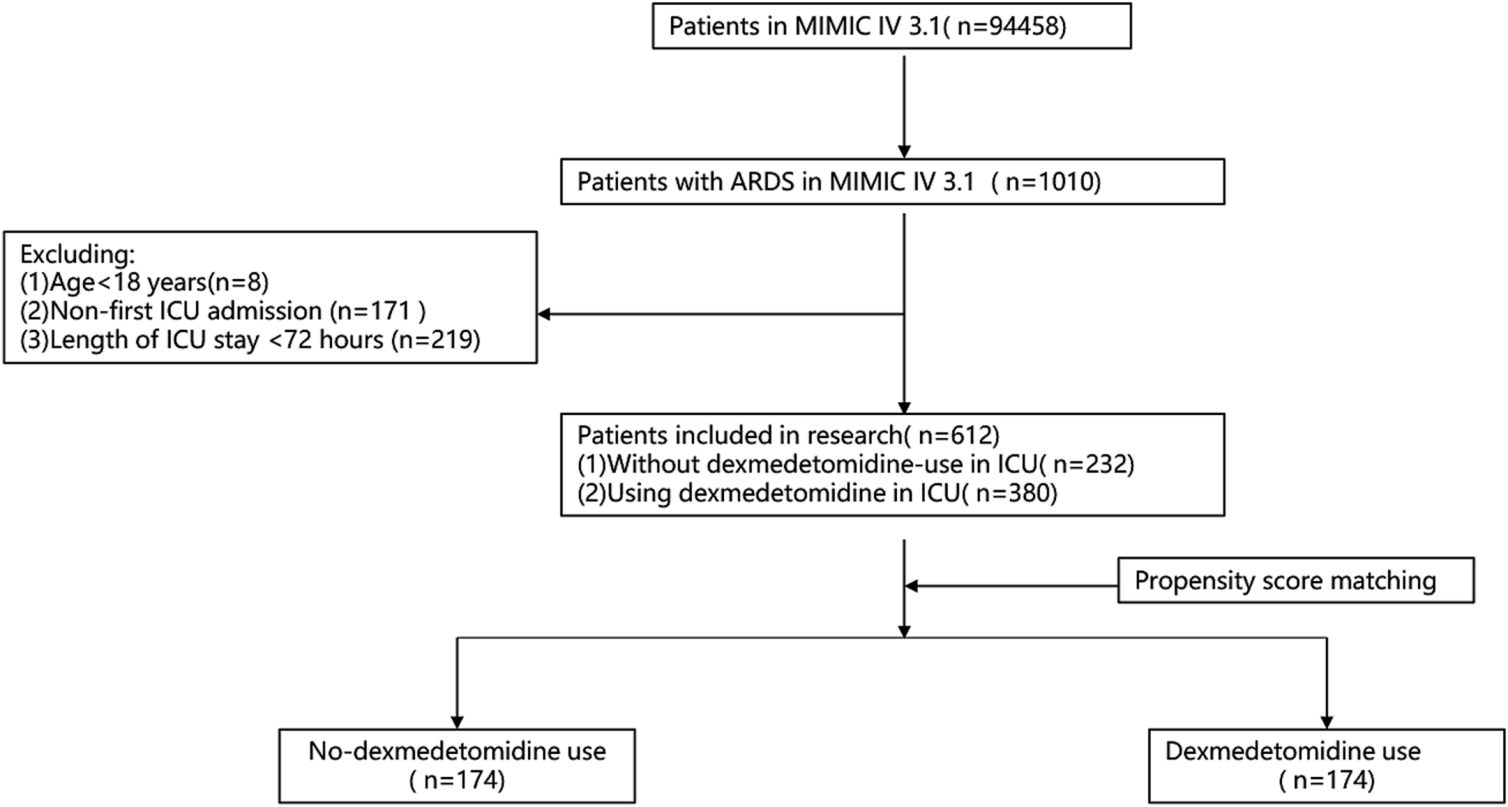
A flowchart illustrating the criteria for inclusion and exclusion.

Data were extracted from the MIMIC-IV database, comprising a cohort of 612 patients, as depicted in Figure 1. Within this cohort, 380 patients (approximately 62.1%) received dexmedetomidine (DEX) during their ICU stay. A detailed breakdown of baseline characteristics for both the DEX-treated group and the non-DEX group is systematically presented in Table 1. It was observed that the DEX-treated patients generally presented with a younger demographic profile and had higher instances of sepsis and mechanical ventilation usage compared to their counterparts. Further analysis through propensity score matching (PSM), as also detailed in Table 1, allowed for a balanced comparison between the two groups. After the application of PSM, 174 patients who were administered DEX were effectively matched with 174 patients from the non-DEX group, ensuring comparability. This matching process confirmed that patient characteristics were evenly distributed between the groups, as all *p* values were found to be above the 0.05 threshold, indicating no significant differences statistically.

### 3.2 Primary outcome

In this investigation, data from the MIMIC-IV database revealed that within a cohort of patients, 56.9% (132 out of 232) of those not treated with dexmedetomidine (non-DEX) experienced mortality within 28 days, a rate significantly higher than the 22.6% (86 out of 380) observed in patients who received DEX treatment during their ICU stay. Statistical tests demonstrated a significant discrepancy with a *p*-value below 0.001. Upon applying propensity score matching techniques, the revised 28-day mortality rate for the cohort administered DEX markedly decreased to 30.5%. This reduction contrasts profoundly with the mortality rate of 60.3% observed in the cohort not receiving DEX treatment. Such findings prominently suggest the potential effectiveness of the therapy (*p* < 0.001), as elaborated in Table 2.

**Table 2:** Primary and secondary outcomes of the study.

Outcomes	Matching	Total	Non-DEX group	DEX group	*p*-value
28-day mortality	Before PSM, *n* (%)	218/612 (35.6)	132/232 (56.9)	86/380 (22.6)	< **0.001**
	After PSM, *n* (%)	158/348 (45.4)	105/174 (60.3)	53/174 (30.5)	< **0.001**
90-day mortality	Before PSM, *n* (%)	255/612 (41.7)	146/232 (62.9)	109/380 (28.7)	< **0.001**
	After PSM, *n* (%)	180/348 (51.7)	114/174 (65.5)	66/174 (37.9)	< **0.001**

DEX, dexmedetomidine; PSM, propensity score matching. Bold values are less than 0.05.

Cox proportional hazards models were used to further quantify the relationship between DEX administration and survival over the 28-day period in the ICU. The findings, as summarized in Table 3, showed that DEX usage significantly reduced the risk of 28-day mortality (univariate model: hazard ratio [HR] = 0.286, 95% Confidence Interval [CI]: 0.218–0.376, *p* < 0.001). The reduction in risk persisted even after adjusting for potential confounding variables (multivariate model: HR = 0.286, 95% CI: 0.214–0.383, *p* < 0.001). A rigorous approach involving 1:1 propensity score matching was undertaken, involving a total of 348 ARDS patients, to confirm these initial results. The beneficial association of DEX with reduced 28-day mortality continued to hold true after this matching, as evidenced by a HR of 0.331 (multivariate model: 95% CI: 0.236–0.463, *p* < 0.001), further detailed in Table 3.

**Table 3:** Association between dexmedetomidine use and in-ICU mortality.

Variables	Univariate model		Multivariate model	
	HR (95% CI)	*p*-value	HR (95% CI)	*p*-value
Dexmedetomidine use				
28-day				
No	Ref		Ref	
Yes	0.286 (0.218, 0.376)	**< 0.001**	0.286 (0.214, 0.383)	**< 0.001**
90-day				
No	Ref		Ref	
Yes	0.314 (0.244, 0.402)	**< 0.001**	0.316 (0.242, 0.412)	**< 0.001**
After PSM				
Dexmedetomidine use				
28-day				
No	Ref		Ref	
Yes	0.375 (0.269, 0.522)	**< 0.001**	0.331 (0.236, 0.463)	**< 0.001**
90-day				
No	Ref		Ref	
Yes	0.414 (0.305, 0.562)	**< 0.001**	0.366 (0.269, 0.498)	**< 0.001**

Univariate model: no adjustment for covariates.

Multivariate model: adjusted age, sex, race, marital status, congestive heart failure, sepsis, hypertension, diabetes, chronic pulmonary disease, acute kidney injury, SOFA score, APS III, heart rate, mean arterial pressure, respiration rate, CRRT, and invasive ventilator administration.

After PSM: Multivariate model adjusted age, sex, race, marital status, congestive heart failure, sepsis, hypertension, diabetes, chronic pulmonary disease, acute kidney injury, SOFA score, APS III, heart rate, mean arterial pressure, respiration rate, CRRT, and invasive ventilator administration.

HR, hazard ratios; CI, confidence interval; PSM, propensity score. Bold values are less than 0.05.

**Figure 2:**
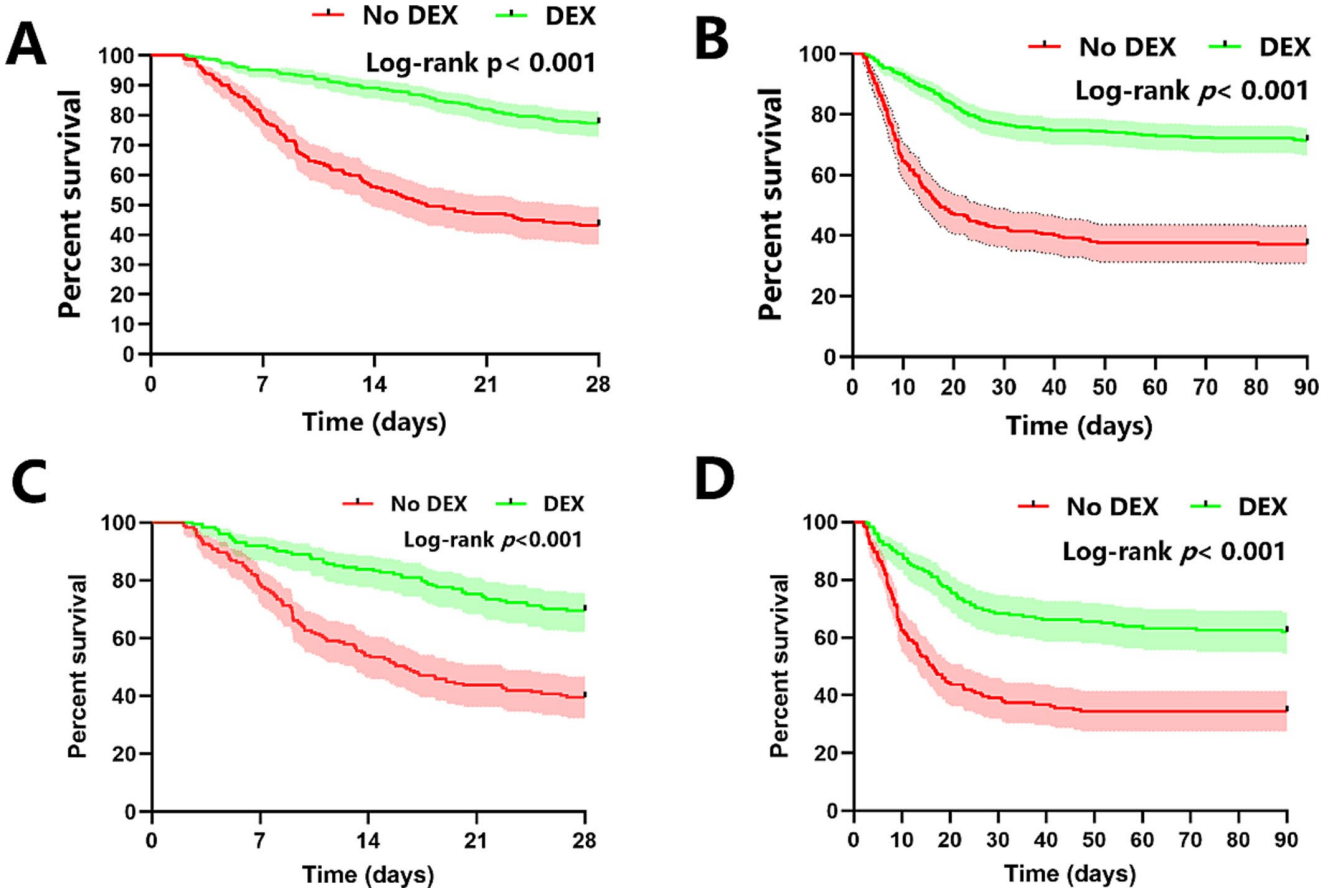
Kaplan–Meier survival curves for 28-day and 90-day in-ICU mortality in ARDS patients from the DEX group and the non-DEX group, both prior to **(A,B)** and subsequent to **(C,D)** PSM.

A Kaplan–Meier survival analysis was executed to further elucidate differences in mortality rates between patient groups. At the conclusion of the 28-day period, the analysis revealed a significantly reduced mortality rate in patients treated with DEX compared to those who did not receive DEX (log-rank test: *p* < 0.001; Figure 2). This analysis visually reinforced the statistical findings, illustrating a clear survival advantage for patients treated with DEX during their ICU admission.

### 3.3 Secondary outcome

The 90-day mortality rate in the DEX cohort was 28.7% (109 of 380), indicating a substantial reduction relative to the 62.9% (146 of 232) recorded in the non-DEX cohort (*p* < 0.001).

Similarly, the results of the univariate Cox regression analysis demonstrated that DEX usage correlated with a reduced risk of 90-day mortality relative to non-use (univariate model: HR = 0.314, 95% CI: 0.244–0.402, *p* < 0.001). The association maintained its statistical significance after adjusting for potential confounding variables (multivariate model: HR = 0.316, 95% CI: 0.242–0.412, *p* < 0.001). The association between DEX administration and 90-day mortality persisted as significant after PSM (multivariate model: HR = 0.366, 95% CI: 0.269–0.498, *p* < 0.001) (Table 3).

A Kaplan–Meier survival analysis demonstrated that the DEX group exhibited a markedly reduced mortality rate relative to the non-DEX group at the 90-day interval (log-rank test: *p* < 0.001; Figure 2).

## 4 Discussion

This retrospective cohort study revealed that patients administered DEX had markedly reduced 28-day and 90-day mortality rates in comparison to those who were not given DEX. Following the adjustment for baseline disparities via propensity score matching (PSM), DEX utilization was consistently linked to diminished mortality rates. Multivariate Cox regression analysis further corroborated the protective effect of DEX on 28-day and 90-day mortality in ARDS patients admitted to the ICU. The Kaplan–Meier survival curves corroborated these findings, indicating extended survival in the DEX group.

The adrenergic system exhibits a significant interrelationship with the immune system (18). Innate and adaptive immune cells express adrenergic receptors, allowing direct responses to signal from the sympathetic nervous system (19, 20). Postganglionic sympathetic nerve fibers, primarily secreting norepinephrine, modulate primary and secondary lymphoid tissues (21). The interplay between the adrenergic and immune systems has garnered heightened interest in recent years. DEX selectively binds with high affinity to α2 adrenergic receptor subtypes α2A and α2C, as indicated in references (22, 23). DEX regulates norepinephrine release by activating α2 receptors on the presynaptic membrane, which supports its immunomodulatory effects. Studies confirm the critical role of DEX in modulating cellular immunity, dampening inflammatory activity within the tissues, and obviously boosting the immune function of patients who are being treated (24, 25). Also, a growing body of research strongly validates that DEX possesses comprehensive protective benefits to a variety of organ systems. The utility of DEX in protection of multiple organs during acute organ injury and the perioperative period, owing to its anti-inflammatory and immunoregulatory properties, has been well investigated (26–28). DEX has also shown its protective properties against diverse organ injuries in several animal models (29–31). In addition, DEX protects against damage to vital organs by blocking ferroptosis in both *in vitro* and *in vivo* experiments (32).

ARDS continues to be a challenge in medical management because of its persistently high mortality. Treatment modalities now place substantial emphasis on supportive care directed to slowing the rate of lung function deterioration and improving survival of the patients. Therapeutic strategies for ARDS include implementing mechanical ventilation to promote breathing, positioning a patient in the prone position for better oxygenation, using neuromuscular blockers to facilitate easier ventilation and applying extracorporeal life support for maintenance of vital functions when other measures do not work (33, 34). Despite these interventions, mortality of ARDS remains at an unacceptable 35% and there is a dearth of effective pharmacologic intervention (35). The complicated pathophysiology of ARDS consists of multiple mechanisms of injury, inflammation, and coagulation in lung and the systemic circulation (1). Therefore, pharmacological interventions suggest a potential strategy for the treatment of ARDS (34, 36). However, the therapeutic function of DEX as a drug rather than a sedation has not been sufficiently investigated in patients with ARDS.

To the best of our knowledge, this is the first investigation of the relationship between DEX administration and in-ICU mortality in patients with ARDS. The results help inform the strategies and clinical management of ARDS therapy. Additionally, through application of multivariate Cox regression and PSM, confounding factors were controlled, and the reliability of the study was strengthened. Yet some limitations exist. First, population data were gleaned from a single center retrospective cohort study which is prone to selection bias, decreasing the generalizability of the results to other ARDS populations. Second, detailed timing and criteria of DEX administration were not available within the MIMIC-IV database, which could have introduced bias into assessing the effect of DEX on reducing mortality. Third, outcomes may have been influenced by unmeasured variables. Fourth, more potential confounders should be included in propensity score matching, such as the etiology of ARDS, the severity of ARDS, variations in treatment strategies, and the duration of mechanical ventilation, PaO2/FiO2 ratio, the mode and settings of invasive ventilator, the use of prone position and ECMO, and the use of other sedatives, analgesics, and neuromuscular blockades, which are demonstrated to influence the clinical outcomes of ARDS (37, 38). However, a large proportion of these factors are missing in MIMIC-IV database, which may cause inaccuracy in statistical results. Thus, we did not include all the confounding factors in propensity score matching. Moreover, because data from the MIMIC-IV database include data from 2008 to 2022, the results may not fully reflect the most recent ARDS management. In the case of this study, patients who developed ARDS early in the ICU stay were excluded, making the applicability of the results to this specific group of patients uncertain. Additionally, changes in dexmedetomidine usage over time, potentially linked to the adoption of other ARDS therapies, represent a potential source of bias. These limitations highlight the need for well-designed, prospective randomized controlled trials to definitively establish the efficacy of dexmedetomidine in ARDS and to address these potential confounding factors. In summary, the findings indicate an association of DEX administration with the better prognosis in ARDS patients. However, the standard therapy for ARDS is still limited to low tidal volume ventilation (LTVV), prone position and neuromuscular blockade. It is crucial to recognize the inherent limitations of a retrospective study design. These findings should be validated by further prospective trials and DEX’s full therapeutic potential in ARDS should be explored.

## 5 Conclusion

In summary, the current research demonstrates that dexmedetomidine administration correlates with reduced 28-day and 90-day mortality rates in ICU patients with ARDS. The potential of dexmedetomidine as a treatment for individuals with ARDS is significant and requires further investigation.

## Data Availability

The original contributions presented in the study are included in the article/Supplementary material, further inquiries can be directed to the corresponding author.
